# Being critical and constructive: a guide to peer reviewing for librarians

**DOI:** 10.5195/jmla.2017.100

**Published:** 2017-01

**Authors:** Katherine G. Akers

As a peer reviewer for a journal, you have an important role to play. You help the editor sift through the myriad of submissions, evaluating the strengths and weaknesses of manuscripts and thereby helping make decisions about which works are important and rigorous enough to be shared with our community of library researchers and practitioners. As in most fields of study, however, librarians usually do not receive formal training in how to review a manuscript. Instead, we may learn how to be a peer reviewer by receiving reviews of our own manuscripts that we have submitted to journals or by picking up general tips and tricks gleaned from our intimate understanding of the scholarly publishing process. Not surprisingly, therefore, peer-reviewing skills are unevenly distributed across librarians, and even experienced peer reviewers may persist in wondering whether they are successfully accomplishing this task.

## DOUBTING YOUR EXPERTISE

If you are relatively new to scholarly publishing and peer reviewing, you may feel a twinge of doubt the first couple of times you are asked to review a manuscript. You may fear that you will not be able to come up with at least one solid criticism of the manuscript that now lies in your hands. Rest assured, however, that this is quite unlikely.

Think of yourself as an external consultant [1]. Realize that you are not expected to be an expert in all aspects of the study described in the manuscript. For instance, you may be well acquainted with the study’s subject matter but not its methodology. This is okay. Comment on what you can, and chances are that the other reviewers of the manuscript will be able to point out issues that you missed (and that you will be able to point out issues that the other reviewers missed). Remember that you have a unique perspective on the work. By virtue of being a person who did not conduct the research or write the manuscript—by having intellectual and emotional distance from the work—you are in an ideal position to spot methodological errors or inconsistencies in reporting, to determine whether the descriptions of the methodology and results make sense, and to judge whether the manuscript advances the knowledgebase or the practice of librarianship.

If you are new to the library profession, realize that your inexperience is not necessarily a detriment to your peer-reviewing ability. In fact, for medical journals, studies consistently show that younger reviewers or reviewers with less professional experience write higher quality reviews than those who are older or have more experience [2–5]. Although it is not yet known why younger reviewers write better reviews, it may be because they are more enthusiastic, less couched in their own methods and opinions, or less burdened by other professional responsibilities.

## THE PROCESS OF REVIEWING A MANUSCRIPT

Some common advice given to peer reviewers is to read the manuscript at least twice [6, 7]. The purpose of the first reading is to get an overall sense of why the study was performed, how it was performed, and what its main findings are. As you read through the manuscript a second time, pay attention to the details of the methodology, the data reported in the text and shown in tables or figures, and the logic of the authors’ arguments and conclusions.

Take notes while you read the manuscript, and then construct those notes into a carefully written review. Discuss both the strengths and weaknesses of the manuscript. When you find a weakness, suggest a concrete way to overcome the shortcoming, if possible: “It’s the easiest thing in the world to poke holes into something. It is usually much harder to suggest how to fix them” [8]. It is usually helpful to the editor and authors if you conceptually organize your review in some manner. For instance, your comments could be grouped into major versus minor points or separated by sections (e.g., Introduction, Methods, Results, and Discussion). Enumerating your comments or using bullet points also helps the editor and authors tease apart multiple concerns. Although identifying individual grammatical errors is generally not advised, it is appropriate to mention if you think the manuscript is poorly written overall or in particular sections. After completing the first draft of your review, read it again to ensure that it is written in a professional tone. Be polite but confident; do not be afraid to point out the manuscript’s limitations.

To get started reviewing a manuscript, consider the following questions.

### Introduction

- Do the authors provide a compelling rationale for why they conducted the study?
- Do the authors clearly describe the purpose of the study and/or state their hypothesis?

### Methods

- Are the methods fully and clearly described?
- Is the methodology sound? Are there potential sources of bias?
- Do the authors utilize objective measures (e.g., of impact, use, success) when possible?

### Results

- Are the results reported in the text consistent with the data shown in the figures or tables?
- Are the statistical analyses performed and reported appropriately?

### Discussion

- Do the authors draw clear conclusions based on the results (as opposed to simply restating the results)?
- Are the conclusions justified by the data (or do they overreach the data)?
- Do the authors explain how their findings advance the knowledgebase and practice of the field?

### Figures and tables

- Do the figures and tables clearly convey the information?
- Are all of the figures and table necessary (or, conversely, do you recommend additional figures or tables)?

### Overall

- Would the manuscript interest the journal’s readership?
- Is the writing straightforward and to-the-point?
- Are there areas of the text that the authors should clarify, elaborate upon, or omit?
- Are the authors missing any pertinent references or body of literature?

There is no set recommendation for how long reviewing a manuscript should take. Although some reviewers may spend 8 or more hours reading the manuscript and writing a review [9], a study of a public health journal shows that completing a review takes 2.7 hours on average [10]. Furthermore, spending more time performing a review does not necessarily result in a stronger review. Whereas one study reports that spending 4 hours or more is associated with higher quality reviews [3], another study reports that there is no further improvement in review quality after 3 hours [4].

Although writing a review need not take a great amount of time, it is generally true that longer reviews are better than very brief reviews. More specifically, a good, comprehensive review should typically be between one-half to two pages in length [11], depending on the complexity and quality of the manuscript. Some sound advice is to “be loquacious” [8]. When you make a suggestion to the authors, explain your reasoning behind that suggestion. Trying to interpret a reviewer’s vague comments can be extremely frustrating for authors. On the other hand, clearly explaining your thinking makes it much more likely that the authors will be able to accurately and adequately address your concerns.

Finally, if you find a “fatal flaw” in the manuscript, such as an error in logic or the use of an inappropriate research design or approach that cannot be remedied by collecting additional data or rewriting the manuscript, it is unnecessary to prepare a long list of minor comments; rather, simply communicate your major concerns [11].

## GETTING SCHOLARLY CREDIT FOR PEER REVIEWING

Conducting peer review is a service to our academic and professional community. Because authors and readers are usually unaware of the identity of reviewers, peer reviewing is mostly a thankless job. However, some recent initiatives aim to provide scholarly credit for peer review (although this is not without controversy [12]). In particular, Publons is a social media site that can verify your review assignments, showcase your peer review contributions, and allow you to keep a record of your reviewing activity for tenure or promotion applications or annual performance reviews [13].

Despite radical changes in scholarly communication, peer review is still considered a necessary process that increases the quality of published research [14]. Speaking on behalf of the editorial team at the *Journal of the Medical Library Association,* we sincerely thank our peer reviewers, who take great care in moderating and strengthening the discourse on the research and practice of health sciences librarianship.
